# Enhancing Biocidal Capability in Cuprite Coatings

**DOI:** 10.1021/acsbiomaterials.2c01222

**Published:** 2023-06-02

**Authors:** Brian T. Lejeune, Xiaoyu Zhang, Su Sun, Julia Hines, Kevin W. Jinn, Ashlyn Neal Reilly, Heather A. Clark, Laura H. Lewis

**Affiliations:** † Department of Chemical Engineering, Northeastern University, 360 Huntington Ave, Boston, Massachusetts 02115, United States; ‡ Department of Mechanical and Industrial Engineering, Northeastern University, 360 Huntington Ave, Boston, Massachusetts 02115, United States; § Department of Bioengineering, Northeastern University, 360 Huntington Ave, Boston, Massachusetts 02115, United States; ∥ Department of Chemistry, Northeastern University, 360 Huntington Ave, Boston, Massachusetts 02115, United States; ⊥ The George J. Kostas Research Institute for Homeland Security, Northeastern University, 360 Huntington Ave, Boston, Massachusetts 02115, United States

**Keywords:** biocidal, antimicrobial coatings, contact-kill, lattice defects, cryomechanical processing

## Abstract

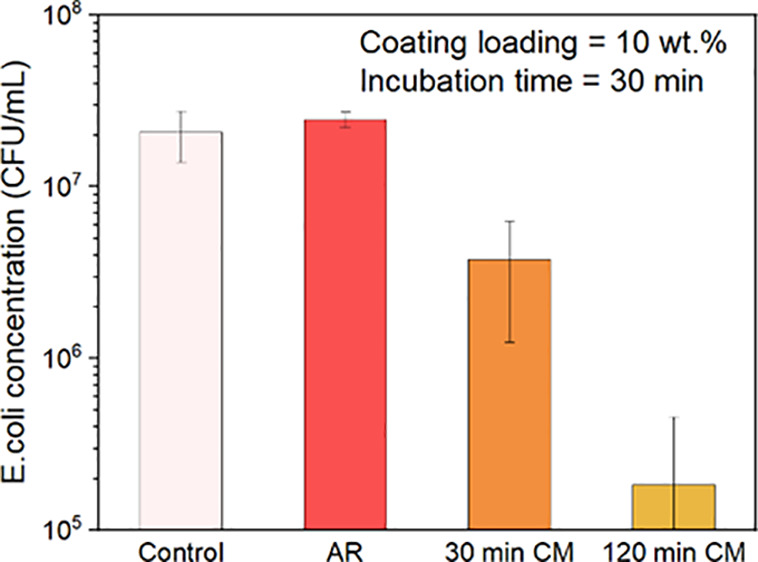

The SARS-CoV-2 global pandemic has reinvigorated interest in the creation and widespread deployment of durable, cost-effective, and environmentally benign antipathogenic coatings for high-touch public surfaces. While the contact-kill capability and mechanism of metallic copper and its alloys are well established, the biocidal activity of the refractory oxide forms remains poorly understood. In this study, commercial cuprous oxide (Cu_2_O, cuprite) powder was rapidly nanostructured using high-energy cryomechanical processing. Coatings made from these processed powders demonstrated a passive “contact-kill” response to *Escherichia coli* (*E. coli*) bacteria that was 4× (400%) faster than coatings made from unprocessed powder. No viable bacteria (>99.999% (5-log10) reduction) were detected in bioassays performed after two hours of exposure of *E. coli* to coatings of processed cuprous oxide, while a greater than 99% bacterial reduction was achieved within 30 min of exposure. Further, these coatings were hydrophobic and no external energy input was required to activate their contact-kill capability. The upregulated antibacterial response of the processed powders is positively correlated with extensive induced crystallographic disorder and microstrain in the Cu_2_O lattice accompanied by color changes that are consistent with an increased semiconducting bandgap energy. It is deduced that cryomilling creates well-crystallized nanoscale regions enmeshed within the highly lattice-defective particle matrix. Increasing the relative proportion of lattice-defective cuprous oxide exposed to the environment at the coating surface is anticipated to further enhance the antipathogenic capability of this abundant, inexpensive, robust, and easily handled material for wider application in contact-kill surfaces.

## 1 Introduction

Creation of durable, cost-effective, and environmentally benign passive contact-kill coatings for frequently contacted (“high-touch”) surfaces is an enduring public health aspiration that has recently escalated with the increased emergence of antibiotic-resistant pathogens, as well as with elevated use of chemical hand sanitizers, especially in light of the SARS-CoV-2 global pandemic.^1−4^ New types of antimicrobial polymers^5−8^ and inorganic surface coatings that evince antipathogenic capabilities can provide alternatives to traditional chemical surface sterilization in public areas. The historic antimicrobial capability of metallic copper and its alloys is well established,^9−14^ and copper-based materials continue to be key components in fungicides and marine antifouling paint.^15−18^ Regulatory agencies have concluded that many copper compounds are relatively safe and do not have adverse effects on animal or human health.^19^ However, while the refractory (i.e., high melting point), low-density, and inexpensive oxide form of copper, Cu_2_O (also known as cuprous oxide or cuprite) has also been documented as an antimicrobial material, origins of its effectiveness are much less well understood. The biocidal activity of Cu_2_O is reported to be elevated at high concentration^20^ or when manifest in potentially hazardous nanoparticulate forms,^21−24^ with higher antipathogenic potential associated with particular crystal lattice planes or facets of chemically synthesized crystallites.^2,20,25−30^ Categorized as a passive (“contact kill”) antimicrobial material,^18,31^ cuprite requires no external energy to activate its biocidal capabilities as does, for example, titania (TiO_2_), which requires UV irradiation.^32−35^ Cuprite is a well-known *p*-type semiconductor with a direct bandgap energy in the range 2.0–2.5 eV (within the visible light spectrum and responsible for its characteristic crimson color) that is tunable by atomic defect engineering, as well as by elemental substitution within its crystal lattice.^36−41^ Surprisingly, the origin of copper oxide’s antimicrobial activity remains controversial, with reactive oxygen species (ROS) and copper ion release cited as contributing factors to varying degrees.^2,15,27,28^

The reported correlations between cuprous oxide’s crystallographic aspects and biocidal activity motivate the closer investigation of its native crystal lattice condition. Indeed, Cu_2_O may be more properly designated as Cu_2−δ_O, where δ quantifies the natural concentration of copper cation vacancies in the lattice per formula unit; δ is reported to be in the range 3.4 × 10^–4^–3.6 × 10^–3^ for cuprous oxide produced from intentionally oxidized pure copper.^31,33−35^ It is known that many transition-metal oxides commonly contain lattice vacancies, also referred to as point defects,^42^ that can donate highly elevated local electrical potential.^43^ By analogy, it is hypothesized that the native cation vacancies in cuprite, and the associated adjustments to the crystal lattice, play a role in its antipathogenic character to disrupt cell membranes and/or virus protein shells. This hypothesis is in alignment with previous study by the current authors who reported that induced lattice defects in titanium oxide nanostructures amplified its catalytic activity.^44^ That finding allowed contemplation that increasing the concentation of lattice defects in cuprite will also increase its antipathogenic potential.

In this current work, we subjected commercial Cu_2_O powders to high-energy mechanical processing to intentionally damage the crystallographic lattice structure. Coatings made from these processed powders demonstrated a significantly enhanced biocidal activity in the presence of *Escherichia coli* (*E. coli*) to achieve a bacterial reduction of >99% achieved upon 30 minutes’ exposure to the coatings. The effects of mechanical processing on the cuprite structure were thoroughly investigated, and analyses of these results suggest that the enhanced contact-kill capability of processed Cu_2_O is indeed derived from induced crystal lattice disorder. This capability may be further enhanced by amplifying the concentration of lattice defects within the cuprite structure.

## 2 Methods

Commercial Cu_2_O powders were systematically processed via cryomilling, a high-energy mechanical processing technique utilized to reduce the physical scale of materials and deliver large amounts of plastic deformation, resulting in significant crystal lattice damage.^45^ This process is conducted at low temperature to minimize thermal recovery of induced lattice damage that would otherwise disappear or “heal” during standard high-energy mechanical milling. It is advantageous that the size of cryomilled powder particles themselves is within the micron range, mitigating potential health risks associated with much smaller nanoparticles.^46,47^ Cu_2_O powders (Sigma Aldrich, >99.99% purity, major impurities iron (6.5 ppm) and barium (4.5 ppm)) were enclosed in Argon-backfilled polycarbonate vials and milled in a liquid nitrogen bath (SPEX Freezer/Mill 6775) at 7.5 min intervals for total cumulative times of 30 and 120 min. All resulting powders were sieved in an inert-atmosphere glovebox to isolate powder size fractions under 25 μm, with the objective to minimize particle aggregation, as well as facilitate the creation of uniform and dense Cu_2_O coatings.

### 2.1 Cu_2_O Powder Characterization

Powder particle morphologies and size distributions were investigated using field-emission scanning electron microscopy (FE-SEM, Hitachi S-4800). Samples were mounted on conductive carbon tape and sputter-deposited with ∼3 nm of Pt to improve the surface conductivity prior to imaging. Crystal lattice aspects of the powders were examined with Cu-K_α_ X-ray diffraction (XRD, PANalytical X’Pert Pro), with phase identification, lattice parameters, and phase fractions determined from the XRD data with Rietveld refinement using the software system GSAS-II.^48^ The lattice microstrain ε and the coherently diffracting crystallite size *D* of the powders were evaluated from the XRD data using the Williamson–Hall approach^49^
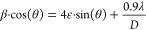
1with β as the Bragg peak full-width at half-maximum, Bragg peak angle θ, and X-ray wavelength λ. The data trend resulting from a plot of β·cos(θ) vs 4·sin(θ) provides an estimate of the average diameter *D* of the crystalline regions, evaluated at the extrapolated *y*-intercept, while the lattice microstrain ε is provided by the data trend slope. The crystallite diameter returned by the Williamson–Hall approach relies upon the degree of crystallinity of a phase, which may not correspond directly with its physical size if noncrystalline regions are present. The characteristic bonding and vibrational modes exhibited by the Cu_2_O lattice were examined at room temperature using Raman spectroscopy (Horiba Jobin Yvon Model HR800) operated in reflection mode in the range 50–1000 cm^–1^, employing an exciting laser beam of wavelength of 532 nm. Data were collected from hand-compacted powder samples.

### 2.2 Cu_2_O Coating Preparation and Physical Assessment

Coatings containing Cu_2_O powders of varied processing states were prepared by embedding powders into the surface of a thin layer of MINWAX fast-drying polyurethane (PU) painted onto to circular glass coverslips. This initial PU layer was allowed to partially cure in air for 9 min, while stable Cu_2_O suspensions were being created by mixing pure isopropyl alcohol (IPA, Sigma Aldrich, >99.7%) with powder in concentations of 1, 2, 5, and 10 wt % and then ultrasonicated for 6 min. A 60 μL droplet of each IPA/powder suspension was deposited to fully cover each partially-cured PU-coated coverslip, which were allowed to dry in air at room temperature for 24 h.

The surface hydrophobicities of both bare PU (control) and fullycured powdercontaining coatings were assessed using optical microscopy to quantify the contact angles of static distilled water drops (10 μL) pipetted directly onto their surfaces. Quantifications were performed in triplicate, with error taken as the standard deviations of the contact angle. Typically, surfaces that exhibit contact angles in excess of 90° are considered to be hydrophobic; in contrast, surfaces exhibiting contact angles less than 90° are categorized as hydrophilic.^50,51^ The cross-sectional and surface particle size profiles of fully-cured coatings were assessed via SEM imaging, with particle size distributions quantified from SEM images using ImageJ software.^52,53^ Data were fit to a two-parameter log-normal probability distribution,^54^eq 2:
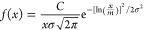
2where *f*(*x*) is the number of particles with a given diameter *x* (microns), *C* is a constant that is proportional to the total surface area occupied by the analyzed particles, σ quantifies the width of the distribution, and *m* is the median particle diameter (microns). Using the resultant fitted parameters σ and *m*, the mean (average) particle diameter <*d*> and its standard deviation Δ*d* were calculated using eqs 3 and 4:
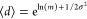
3
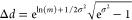
4

### 2.3 Biocidal Assessment of Cu_2_O Coatings

Biological assays were performed to assess the viability of *E. coli* bacteria grown on the surfaces of the Cu_2_O-containing coatings, with polyurethane-coated coverslips utilized as a negative control. Our procedure was modeled on those described in ISO 22196,^55^ which is the current industry standard for antimicrobial surface assessment for plastics and nonporous surfaces.^56^ After transferring one colony-forming unit (CFU) into Mueller–Hinton broth, the *E. coli* bacteria were incubated at 37 °C overnight. Subsequently, the bacteria were passaged into fresh media, incubated again (37 °C) for 1 h, and then diluted to a concentration of 7-log10 CFU/mL in a sterile 0.9% sodium chloride saline solution. One coated and cured coverslip was placed into each well of a six-well culture plate and inoculated at room temperature with 50 μL of the 7-log10 dilution; the coverslips were covered with a 12 mm × 12 mm square Parafilm coupon to prevent evaporation. After bacterial exposure (from 10 min to 24 h), the inoculum was recovered by placing the Parafilm-covered coverslip into 10 mL of saline solution and agitated on a vortex mixer for 30 s. One milliliter of the resultant agitated solution was serially diluted and plated for CFU counting to determine the bacterial burden as compared to stock solution. All bacterial assays were performed in quadruplicate, with error bars representing the standard deviation of the determined CFU.

## 3 Results

Results are reported on Cu_2_O powders and coatings in three unique conditions: the as-received (AR) state, the 30-minute cryomilled (30-min CM) state and 120-minute cryomilled (120-min CM) state.

### 3.1 Effects of Processing on Cu_2_O Powders

**Figure 1:**
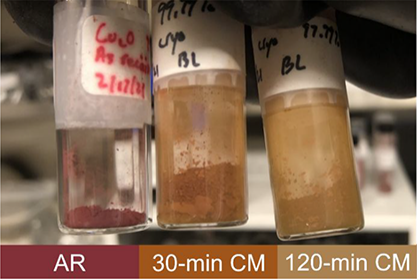
Cu_2_O powders show a processing-induced progressive color change from dark red (AR) to orange (30-min CM) and to yellowish brown (120-min CM).

While the AR (unprocessed) Cu_2_O powder is dark red in color, cryomilling induces a color change to orange after 30 min, which transitions to yellowish brown after 120 min, as shown in Figure 1.

**Figure 2:**
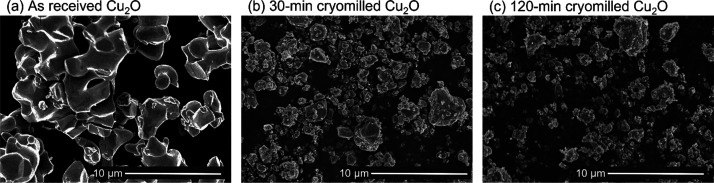
SEM micrographs of (a) as-received (AR) Cu_2_O powder, (b) 30-minute cryomilled powder and (c) 120-minute cryomilled powder.

Electron microscopy revealed that the AR Cu_2_O powder consisted of micron-sized clusters (5–25 μm) formed from multiple smaller, rounded particles of a typical diameter of 2–3 μm. After cryomilling, the particle size is reduced and an irregular angular morphology is acquired, as shown in Figure 2. This particle size reduction stabilized after 30 min of milling time.

**Figure 3:**
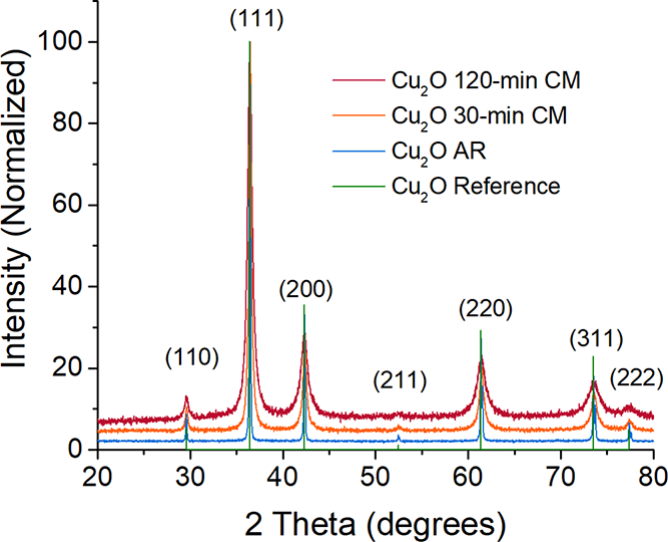
X-ray diffraction data collected from the Cu_2_O samples show increased Bragg peak broadening with increased cryomilling time. Green vertical lines labeled with Miller indices mark (*hkl*) the locations of the major Bragg diffraction peaks of cuprites’s cubic crystal structure.

**Figure 4:**
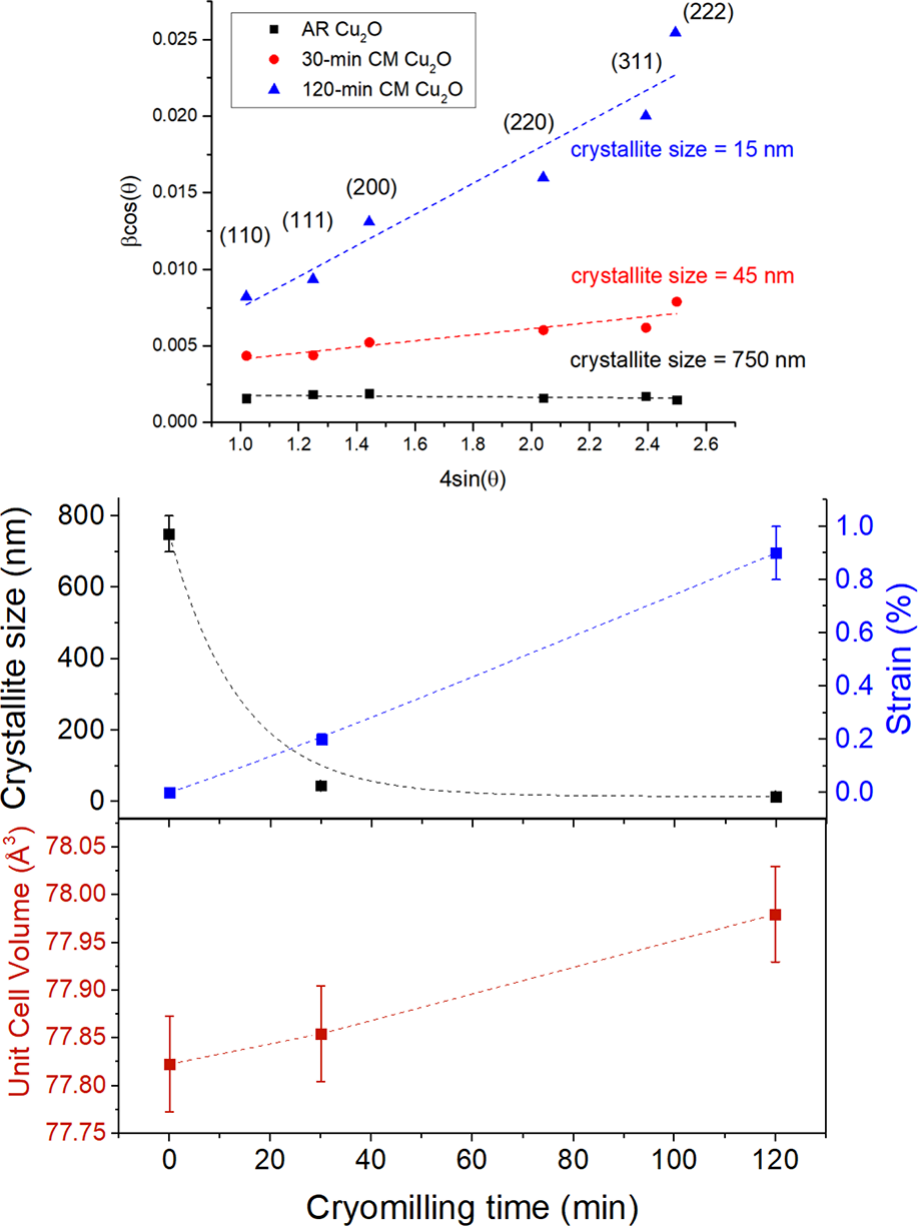
Results derived from X-ray diffraction data. Linear data trends (top) produced by the Williamson–Hall treatment (eq 1) of data confirm an increased microstrain ε level and decreased crystalline size with increased cryomilling time (middle). An increase in the unit cell volume (bottom) is produced by increased cryomilling time. In some cases, error bars are smaller than the size of the data markers and are thus not visible.

Figures 3 and 4 display data and analyses regarding the state of the cuprite lattice, as derived from the XRD data. All Cu_2_O samples exhibited Bragg diffraction peaks corresponding to the cubic *Pn*3̅*m* crystal structure reported for cuprite.^57^ Cryomilling produced a small increase in the Cu_2_O cubic *a*-lattice parameter (*a*(AR) = 4.269(1) Å; *a*(120-CM) = 4.272(1) Å) resulting in a small but statistically significant 0.2% increase in the unit cell volume. Analyses based on the application of eq 1 to the diffraction data revealed that the crystalline particle size decreased exponentially with increased cryomilling time, from ∼750 nm in the AR state to ∼15 nm after 120 min, while the corresponding lattice microstrain values increased linearly from essentially zero to approximately 1%. An absolute lattice strain of 1% is very large, especially as oxide compounds do not typically sustain plastic deformation.

**Figure 5:**
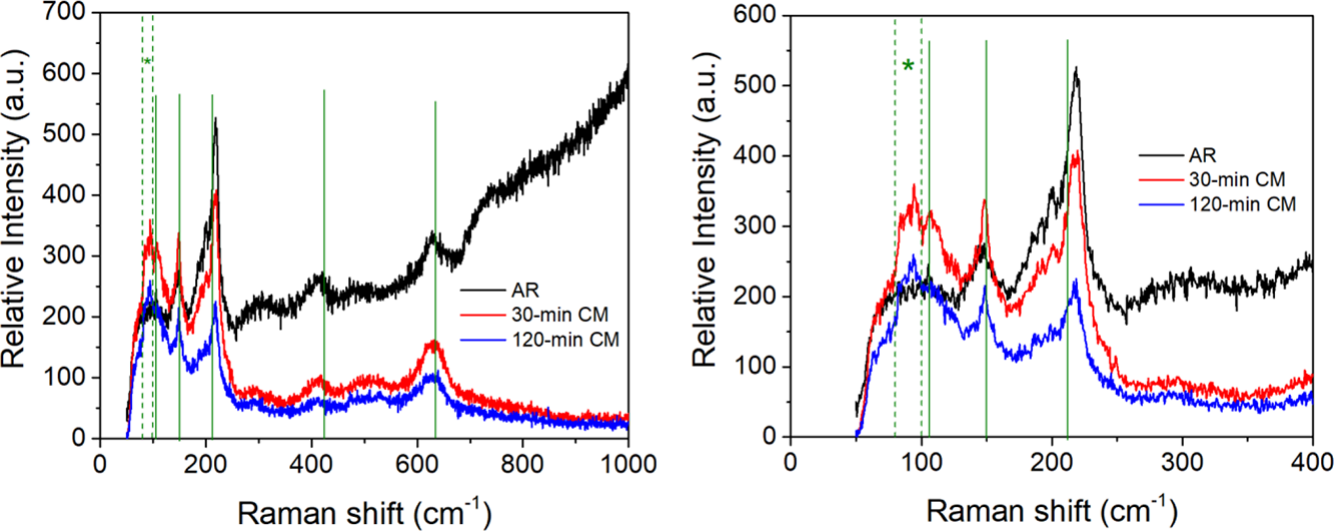
(Left) Raman spectra of the Cu_2_O powder in different processing states, with characteristic phonon frequencies identified by vertical lines at 106, 150, 212, 424, and 635 cm^–1^. A peak in the range 90–100 cm^–1^, observed only in cryomilled samples, is indicated by dashed lines and an asterisk. The increased background intensity noted at higher inverse frequencies for the as-received powder is attributed to the high surface roughness of the powder compact. (Right) An enlarged view of the 0–400 cm^–1^ range of the Raman data.

Raman data documented the evolution of the characteristic Cu_2_O lattice phonon vibrational frequencies with increased processing time, as shown in Figure 5. All spectra displayed similar excitation peaks (106, 150, 212, 424, and 635 cm^–1^) consistent with those reported for micron-sized Cu_2_O particles.^16,58,59^ A general decrease in the characteristic Raman peaks’ sharpness and intensities with increased cryomilling time was noted, and a broad, poorly-formed peak emerged in the region 90–100 cm^–1^ in the cryomilled powder data, which was not observed in data collected from the AR sample.

### 3.2 Physical Character of the Cu_2_O Coatings

**Figure 6:**
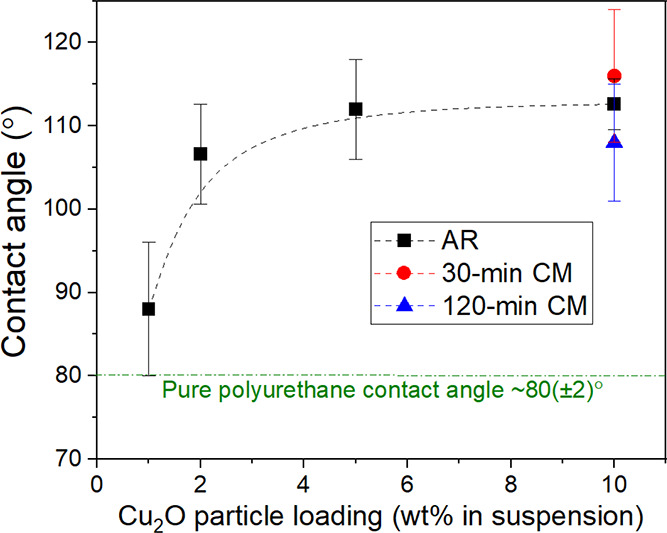
Measured contact angles of distilled water on Cu_2_O coatings as a function of particle loading (Cu_2_O powder weight percentage in IPA to create a suspension). An increase in contact angle, and hence hydrophobicity, with increased particle loading is noted from 88 ± 8° (1 wt % suspension) to 112 ± 4° (5 wt % suspension). The dashed line is provided to guide the eye.

The hydrophobicity of the Cu_2_O coatings, represented by the surface contact angle of distilled water, increased monotonically with increased particle loading, as shown in Figure 6. The hydrophobicity of coatings made from 10 wt % powder suspensions were all similar and were all hydrophobic, whether containing the AR, 30-min CM or 120-min CM powder. In contrast, the pure polyurethane coating exhibited hydrophilic behavior.

**Figure 7:**
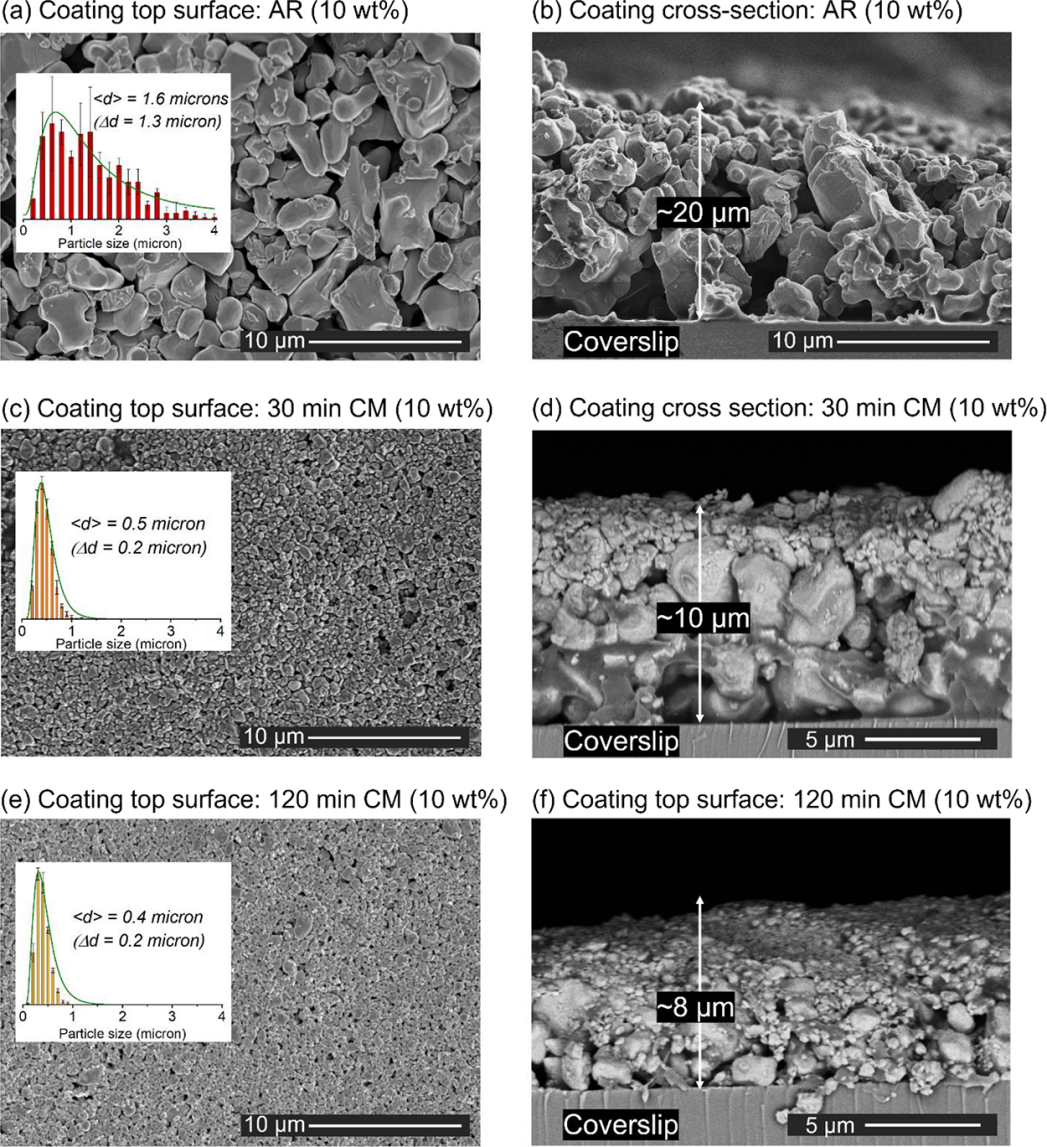
SEM micrographs of Cu_2_O coatings made from 10 wt % Cu_2_O-IPA suspensions. Top-surface views: (a) AR Cu_2_O, (c) 30-min CM Cu_2_O, and (e) 120-min CM Cu_2_O. Particle diameter distribution profiles are provided in the insets, with the average particle diameter (<*d*>) and standard deviation (Δ*d*) calculated based on eqs 3 and 4. Cross-sectional views: (b) AR Cu_2_O, (d) 30-min CM Cu_2_O, and (f) 120-min CM Cu_2_O. The results confirmed uniform, dense particle arrangements on the coatings’ surfaces with smaller particles preferentially segregated to the tops of the coatings.

As evidenced in Figure 7, coatings produced from IPA/10 wt % Cu_2_O suspensions all exhibited dense arrangements of particles that were uniformly distributed on the surface but were highly nonuniform along the cross-section, with smaller particles preferentially segregated at the top. The coating thickness was approximately 20 μm when prepared with the AR Cu_2_O and was reduced to 10 μm for the cryomilled powders. The distributions of the diameters of the surface particles displayed log-normal-like profiles, with average diameters that decreased with an increased processing time from 1.6 ± 0.8 μm (in the AR state) down to 0.5 ± 0.2 μm ((in the 30-minute cryomilled state), only slightly decreasing further to 0.4 ± 0.2 μm in the 120-minute cryomilled state.

### 3.3 Biocidal Response of Coatings

#### 3.3.1 Coatings Made from AR (Unprocessed) Cu_2_O Powder

**Figure 8:**
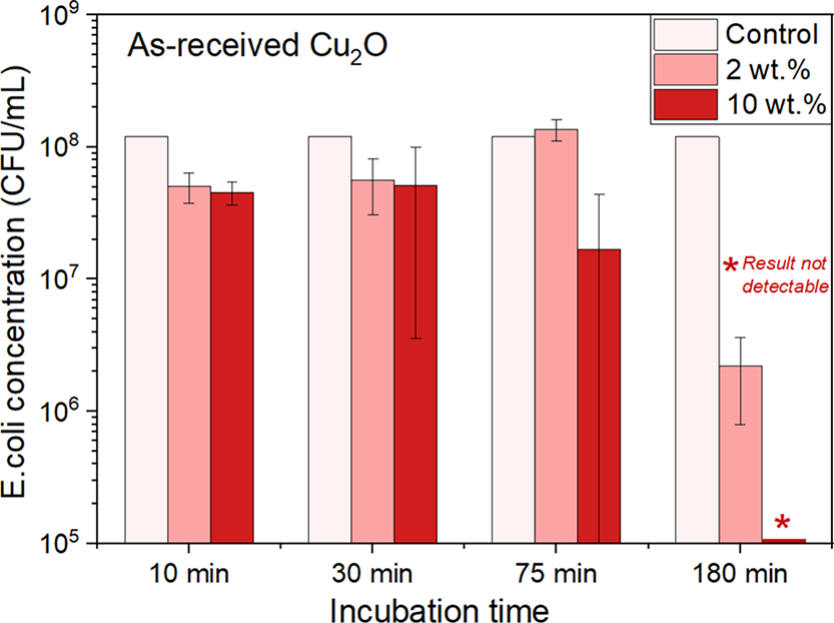
Biocidal time-kill assay results for coupons coated with suspensions of as-received Cu_2_O in IPA (2, 10 wt % concentrations), relative to results obtained from control samples of pure polyurethane (PU). Incubation time refers to the length of time that *E. coli* inocula were left on the coatings’ surface prior to recovery. Only a minimal biocidal response is observed for *E. coli*-inoculated coatings that were incubated for less than 75 minutes, relative to the PU control. The coating made from the IPA/10 wt % suspension exerts biocidal activity upon after 75 minutes of incubation with a completely sterilized coating achieved after 180 minutes of exposure.

Figure 8 depicts baseline time-kill results obtained from coatings made from the AR powder in IPA suspension concentrations of 2 and 10 wt % for four different time points: 10, 30, 75, and 180 minutes. The pure PU control sample did not demonstrate any decrease in *E. coli* viability at all time points tested; its reference value of 1.2 × 10^8^ CFU/mL is included in Figure 8. A noticeable biocidal response against *E. coli* developed after 75 minutes of exposure time to the coating made with the IPA/2 wt % AR powder suspension, with a 2-log10 (100×) reduction in bacterial viability (99% biocidal effectiveness) achieved after 180 min.

A 1-log10 (10×) reduction in bacterial viability was noted between 30 and 75 min of incubation time upon exposure to the coating made from the IPA/10 wt % AR powder suspension. Zero bacteria were detected after 180 minutes of exposure time to coatings of AR Cu_2_O powder, consistent with a greater than 5-log10 reduction in viable bacteria (i.e., 99.999% biocidal effectiveness).

**Figure 9:**
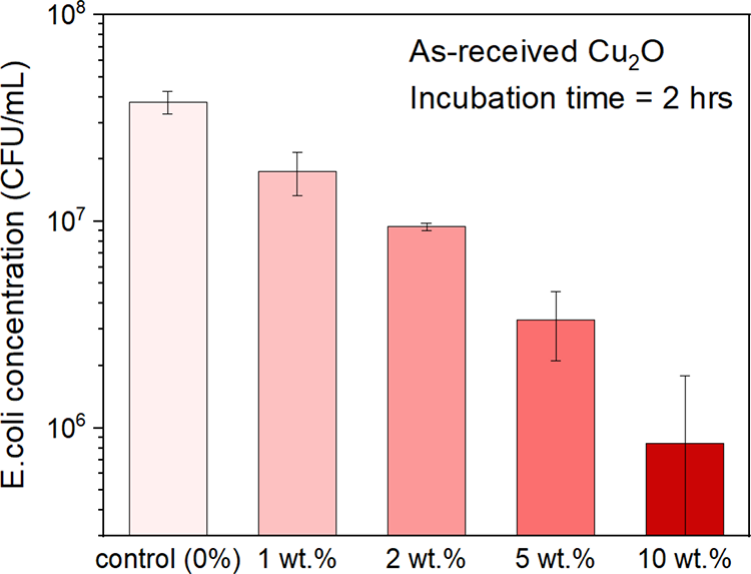
A systematic decrease in bacterial viability with increased Cu_2_O concentration (listed as wt % Cu_2_O powder in the IPA/powder suspension) was observed with a maximum 1.5-log10 (∼50×) reduction in viable bacteria under a 120 min incubation time.

Targeting a constant incubation time point of 120 min, Figure 9, a systematic decrease in viable bacteria with increased concentration of AR Cu_2_O powder in the IPA suspensions was noted, ranging from 3.8 × 10^7^ CFU/mL for the pure PU control to 8.5 × 10^5^ CFU/mL for the coating made with the IPA/10 wt % AR powder suspension, translating to a 1.5-log10 (∼50×) reduction in viable bacteria.

#### 3.3.2 Coatings Made from Cryomilled (Processed) Cu_2_O Powder

**Figure 10:**
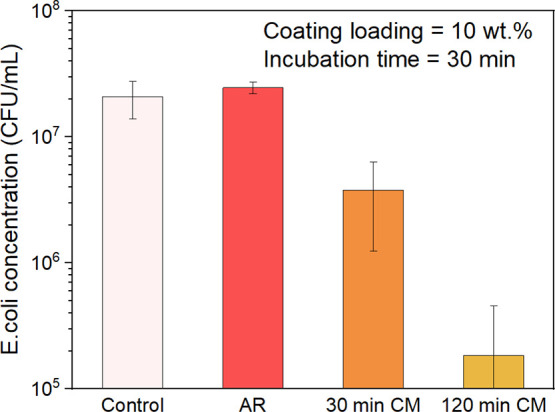
Bacteria viability after 30 min incubation of *E. coli* on Cu_2_O coatings made with 10 wt % IPA/Cu_2_O powder suspensions. At this incubation time, AR Cu_2_O coatings show no biocidal effectiveness, whereas coatings made from the 30-min CM Cu_2_O powder shows a 1-log10 (10×) reduction in viable bacteria, and those made from 120-min CM powder show a 2-log10 (100×) reduction.

Similar investigations were also conducted on coatings made from processed IPA/powder suspensions. Figure 10 documents representative results obtained within 30 minutes of exposure time to coatings made from suspensions of IPA/10 wt % processed powder (both 30-min CM and 120-min CM). It can be seen that coatings made with 30-min CM powder delivered ∼10× decrease in bacterial viability, while a bacterial reduction in excess of 2-log10 (100×, >99%) was observed using coatings made with the 120-CM powder over the same time of exposure. No decrease in viable bacteria, relative to the PU control, was detected upon exposure to the coating made from the unprocessed, AR powder in this condition.

## 4 Discussion and Conclusions

The results presented here indicate that high-energy mechanical processing swiftly and systematically modifies commercial Cu_2_O powder to alter its color, reduce its particle size, and degrade its crystallinity while simultaneously significantly increase lattice microstrain. At the same time, a notably improved contact-kill capability is documented for coatings made from modified powders relative to those made from AR powders. The combination of these two observations allows a causal relationship to be drawn between induced lattice imperfection in Cu_2_O and intensification of its biocidal character in the presence of *E. coli* and suggests routes for further improvement of Cu_2_O-based antimicrobial coatings for application in high-touch surfaces.

The processing-induced color changes noted in the Cu_2_O powders, Figure 1, are consistent with an increase in the bandgap energy permitting the absorption of shorter wavelengths, providing an immediate visual cue that cryomilling is altering more than just the physical size or morphology of the powders. Indeed, while the average powder particle diameter plateaus at ∼1 μm after only 30 minutes of cryomilling, the average dimension of well-crystallized regions within the powder particles continues to decrease with additional processing time, stabilizing at ∼15 nm after 60 minutes of processing. Further, the Cu_2_O atomic lattice itself continues to accumulate damage to reach a surprisingly large microstrain value of 1% after 120 minutes of cryomilling (Figure 4). While this value, which does not appear to saturate, is in alignment with the 1.5% microstrain value reported for thin composite films of Cu_2_O nanoparticles embedded in an amorphous copper oxide matix,^60^ it is well in excess of the 0.5% value reported for FeNi metallic powders conventionally milled for 400 hours.^61^ More specific indications of the type of damage sustained by the Cu_2_O lattice are provided by the Raman spectra, as shown in Figure 5. Systematic reductions in characteristic peak intensity and sharpness with increased processing time correspond to a general weakening of Cu–O bonds and bond angles. Further, as reported by Shi *et al.*, the Raman peak located in the range 90–100 cm^–1^ that emerges upon prolonged processing is associated with an increased population of Cu vacancies within the Cu_2_O lattice.^58,59^ As these vacancies are reported to be energetically favorable, they are likely to develop as a result of high-energy processing. Overall, these data strongly suggest that cryomilling creates nanoscaled crystalline regions dispersed throughout micron-scaled, poorly crystallized individual Cu_2_O powder particles. As the cryogenic processing conditions utilized in this study prevent appreciable atomic diffusion, both the crystalline and the amorphous components of the processed particles are anticipated to posseses the same composition as that of the AR powder, with a slightly expanded lattice parameter reflective of the extensive induced lattice damage.^62^

When mixed with polyurethane, the cryomilled Cu_2_O powders form coatings that are dense and hydrophobic, desired traits for antimicrobial surfaces—if microbes cannot adhere to a surface, they cannot contaminate it. The literature indicates that certain formulations of polyurethane can exhibit hydrophilic or hydrophobic behavior.^63^ Further, literature reports describe both hydrophobic^64^ and hydrophilic^65^ Cu_2_O behavior. Overall, it is noted that surface roughness can also have a profound effect on the measured contact angle of the material based on whether the material is hydrophobic (increases contact angle) or hydrophilic (decreases contact angle). The dramatic difference between the hydrophilic nature of the bare polyurethane surface and the hydrophobic nature of the Cu_2_O-containing coatings, as shown in Figure 6, is consistent with good particulate exposure. This conclusion is supported by micrographs of the coated coverslips (Figure 7) that confirm segregation of smaller and presumably more lattice-defective Cu_2_O particles to the surface of the coatings, displacing larger particles. This segregation is consistent with that observed for cuprous oxide coatings reported by Behzadinasab *et al.*;^20,66^ however, unlike their results, the biocidal capability of our current coatings did not need activation with energy-intensive argon plasma etching or with elevated-temperature thermal treatment, processes that are challenging to apply to irregular or large objects.

Our current results confirm that the amplification of the contact-kill capability of Cu_2_O-based coatings is positively correlated both with the concentration of the incorporated powder, as well as with the processing time and hence with the degree of damage to the Cu_2_O lattice. Coatings made from the highest concentration suspensions (IPA/10 wt %) of AR powder delivered a one-hundred-thousand-fold (5-log10) reduction in bacterial presence relative to the 99% (2-log10) reduction documented from the coating made with the much lower IPA/2 wt % powder suspension, both under long (180-minute) incubation times (Figure 8). Substitution of cryomilled powders for the AR powders greatly accelerated the biocidal response: coatings made with 120-min CM powder required only 30 min to achieve >99% (2-log10) reduction in viable bacteria as compared to those made from the AR powder of the same suspended concentration, which required more than two hours to attain this same reduction, Figure 10. This result translates to a 400% faster passive contact-kill response from coatings made with cryomilled powders. Further, full sterilization—zero detected viable bacteria—was achieved after two hours of exposure to coatings made from the 120-min CM powder (data not shown).

The origin of the enhanced biocidal activity in the presence of *E. coli* found for coatings made from processed powders is attributed to effects resulting from the disruption of the regularity of the Cu_2_O crystalline lattice, along with the likely formation of a large population of energetically favorable cation vacancies.^38,39,41^ These atomic-level aspects produce changes in the Cu–O bonding character which, in turn, are expected to impact the band gap and the maxima/minima in the electronically active 3*d*-like density of states, as well as influence the degree of nonstoichiometry and associated density of local charge states in the cuprite lattice.^67−69^ Lattice-defective Cu_2_O is likely to be concentrated at the surfaces of the smaller particles that have segregated to the top surface of the coatings, where they would be in direct contact with the pathogen-containing environment. Data confirm that longer cryomilling times escalate the lattice microstrain and reduce the Cu_2_O crystallinity but do not greatly impact the average particle size, thereby ruling out a simple increase in the Cu_2_O surface area as the source of the amplified biocidal activity.

These results clarify strategies to further increase the contact-kill effectiveness of coatings incorporating lattice-defective Cu_2_O powder. Practical engineering tradeoffs must be considered to minimize both the powder concentration in the coating and the cryomilling time, while at the same time maximize the contact-kill capability of powder-incorporated coatings. One approach to boost the biocidal effectiveness of these coatings is to extrinsically increase the concentration of Cu_2_O lattice disorder and defects by subjecting the powder to more energetic and/or to longer mechanical processing times. This approach is justified by the documented increase in lattice microstrain that remains linear up to the longest processing time of this study, 120 minutes (Figure 4). Another approach is to intrinsically modify the Cu_2_O lattice character and bond strength through a “defect chemistry” approach in which the introduction of small concentrations of foreign atoms of different atomic radii and/or different valence states to the Cu_2_O host lattice may multiply atomic defects to ensure overall charge neutrality, among other effects.^43,70^ These approaches represent some avenues of future inquiry.

In summary, significant enhancements in the biocidal effectiveness of commercially available Cu_2_O may be achieved readily and rapidly through the application of severe mechanical deformation, which multiplies crystal lattice damage and lattice microstrain. Coatings made from the widely accessible, copper oxide compound Cu_2_O (cuprous oxide, cuprite) were shown to be biocidal in the presence of *E. coli*, and based on previous studies it is likely that these specially processed materials may be effective against other strains of bacteria. Future studies are planned to confirm the gram-negative and gram-positive properties and the full spectrum of biocidal properties. One of the interesting properties of bacteria is their ability to form biofilms on surfaces, which make them even harder to eliminate. Although our current studies did not progress to the biofilm stage, biofilm inhibition assays will be the focus of future studies as we explore the universal biocidal nature of these films. Cuprite surfaces provide a passive (“contact kill”) option to our present antibacterial arsenal, an asset in combating the rise in antimicrobial resistance among potential pathogens. In this current study, Cu_2_O particles were encapsulated in polyurethane, which is the basis of many paints and lacquers and is considered to be robust. For the translation of the study to a field-setting, future study would need to consider the thermal, mechanical, and chemical stability of the particles in the coating.
